# Glucagon-Like Peptide-1 Agonist Use in Adults With Congenital Heart Disease: Effect, Safety, and Outcomes

**DOI:** 10.1016/j.jacadv.2025.101674

**Published:** 2025-03-24

**Authors:** Rashmi Thapa, Kyla M. Lara-Breitinger, Francisco Lopez-Jimenez, Nishat Shama, Alexander C. Egbe, William R. Miranda, Heidi M. Connolly, C Charles Jain, Maan Jokhadar, Angela M. Kosec, Svea Alm, Luke J. Burchill

**Affiliations:** a Department of Cardiovascular Diseases, Mayo Clinic, Rochester, Minnesota, USA; b Department of Physiology and Biomedical Engineering, Mayo Clinic, Rochester, Minnesota, USA

**Keywords:** adult congenital heart disease, GLP-1 receptor agonist, weight loss

## Abstract

**Background:**

Obesity is prevalent among patients with adult congenital heart disease (ACHD) and contributes to adverse cardiovascular outcomes. There is a paucity of data regarding glucagon-like peptide-1 receptor agonists (GLP-1 RA) for weight loss in patients with ACHD.

**Objectives:**

The purpose of this study was to assess the effect, safety, and outcomes of GLP-1 RA among patients with ACHD.

**Methods:**

This is a retrospective cohort study of patients with ACHD at Mayo Clinic (01/2013-01/2024) who were prescribed semaglutide or liraglutide. The primary endpoint was weight loss. Secondary endpoints were changes in New York Heart Association class, hemoglobin A1c, estimated glomerular filtration rate, and safety endpoints of renal adverse event, hypoglycemia, hospitalization/drug discontinuation due to side effects.

**Results:**

Seventy patients received GLP-1 RA over a mean duration of 21 ± 20 months. Majority (85.7%) had moderate/severe complexity congenital heart disease. Weight loss >5% was achieved in 30 (42.9%) patients. Patients with body mass index ≥35 kg/m^2^ were more likely to achieve weight loss >5% [66.7% vs 40%, *P* = 0.027]. Younger age resulted in improved weight loss of 0.17 kg per 1-year age difference (*P* = 0.014). Hemoglobin A1c lowered by a mean of 0.6% (*P* = 0.054). There were no significant changes in New York Heart Association class or estimated glomerular filtration rate. One-third of patients experienced side effects, mostly from gastrointestinal intolerance (20%); 11.4% discontinued the medication due to side effects.

**Conclusions:**

GLP-1 RAs are safe and effective for weight loss in patients with ACHD with beneficial effects on glycemic control.

## Background

As more patients with congenital heart disease (CHD) survive to adulthood, obesity has emerged as a major health concern.^1^^,^^2^ In patients with adult congenital heart disease (ACHD), obesity is associated with an increased risk of adverse events including heart failure hospitalization, heart transplant, and all-cause mortality.^3^ Studies have also shown an increased incidence of diabetes among patients with ACHD with increased mortality compared to diabetics without CHD.^4^^,^^5^

The glucagon-like peptide-1 receptor agonists (GLP-1 RA) semaglutide and liraglutide have favorable effects on cardiovascular outcomes in patients with type 2 diabetes mellitus, especially among those with a high cardiovascular risk profile.^6^, ^7^, ^8^, ^9^, ^10^ The beneficial effects of GLP-1 RA have recently been shown to extend to obese patients without diabetes and those with congestive heart failure.^11^, ^12^, ^13^, ^14^

Currently, there is a paucity of data about the clinical efficacy and benefits of GLP-1 RA in patients with ACHD. The purpose of this study is to determine whether GLP-1 RAs are tolerated, safe, and effective for weight loss in patients with ACHD.

## Methods

This is a retrospective cohort study, in which data were collected by chart review of electronic medical records. The study population includes patients with ACHD with and without diabetes receiving care at Mayo Clinic between January 2013 and January 2024, who were prescribed either semaglutide or liraglutide, regardless of the indication for the drug. GLP-1 RAs not approved for weight loss were not included in the study. The study was approved by the Mayo Clinic Institutional Review Board and received proper ethical oversight. Patients with incomplete data, history of bariatric surgery, or those who took GLP-1 RA for <3 months were excluded.

Baseline clinical characteristics were obtained from data available prior to initiation of the GLP-1 RA. Additional variables were collected as required for the purpose of the study. Obesity was classified according to body mass index (BMI) per World Health Organization guidelines as class I (BMI 30-34.9 kg/m^2^), class II (BMI 35-39.9 kg/m^2^), and class III (BMI ≥40 kg/m^2^).^15^ Temporal changes in weight, GLP-1 RA use, and pertinent clinical parameters were evaluated until drug discontinuation or the most recently available clinic encounter. The primary outcome was weight loss measured as a change in absolute body weight and BMI. The secondary outcomes were: 1) Δ NYHA functional class, 2) Δ hemoglobin A1c (HbA1c), and 3) Δ estimated glomerular filtration rate (eGFR).

The safety endpoints were: 1) renal adverse event defined as >50% reduction in eGFR from baseline; 2) fasting hypoglycemia defined as fasting blood sugar <70 mg/dL; 3) major hypoglycemic event defined as hypoglycemia requiring corrective action; 4) hospitalization due to side effects; 5) discontinuation due to side effects.

### Statistical analysis

Continuous variables are presented as mean ± SD and categorical variables presented as counts (%). Chi-square test was utilized to compare categorical variables, where applicable. Temporal changes were assessed utilizing paired *t*-test at baseline (prior to GLP-1 RA initiation) and at the last date of clinic review, or until GLP-1 RA was stopped. Temporal changes in study indices were reported as an absolute change from baseline and expressed as 95% CIs. Linear regression was used to assess the potential confounding effect of age and sex on the primary outcome. McNemar test was used to determine temporal difference in NYHA functional class. All statistical analyses were performed with Statistical Package for Social Sciences (SPSS) software (version 28.0.0.0, IBM, SPSS Inc), and a *P* value <0.05 was considered statistically significant.

## Results

**Figure 1:**
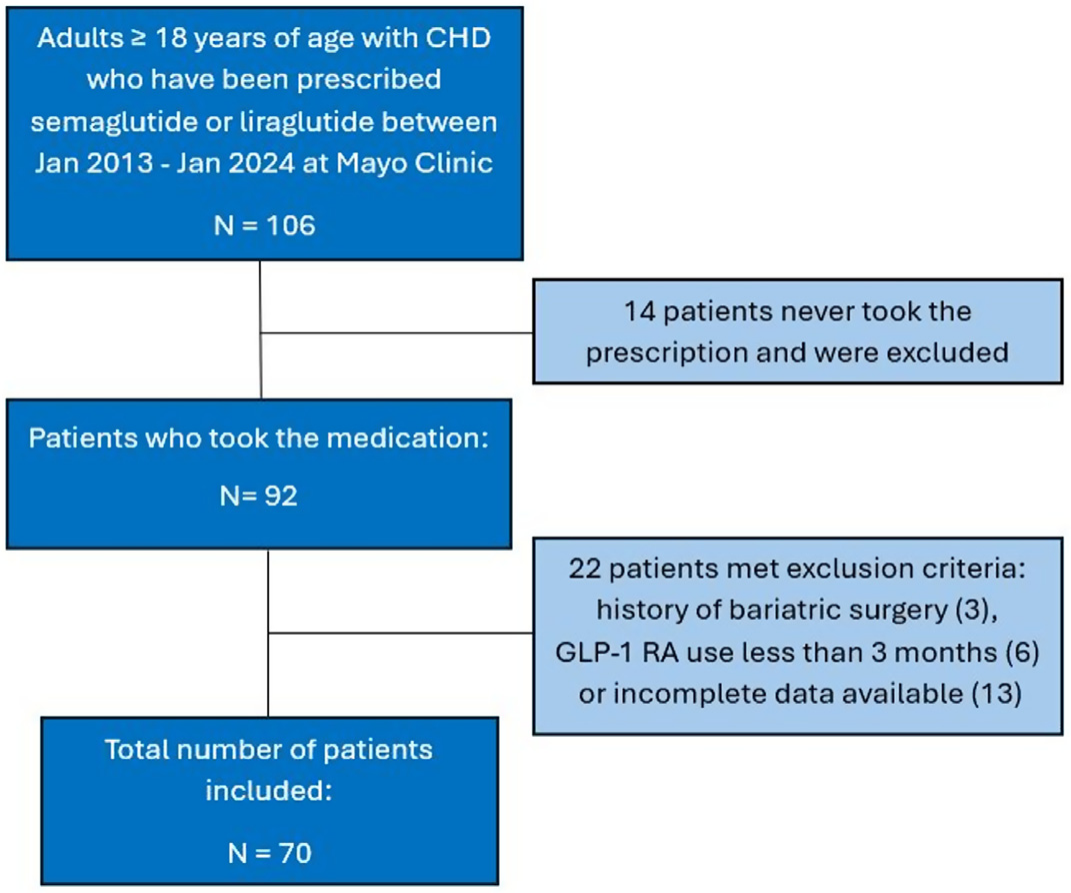
**Inclusion of Study Patients** This figure depicts the inclusion of 70 patients with adult congenital heart disease (age ≥18 years) for the current study. CHD = congenital heart disease; GLP-1 RA = glucagon-like peptide-1 receptor agonist.

A total of 106 patients with ACHD were ever prescribed semaglutide, liraglutide, or both. Of these patients, 14 (13.2%) never took the medication. Reasons for not taking the medication included lack of insurance approval (N = 7), high cost of medication (N = 1), and the prescribed drug not being available (N = 1). The reason for not taking was unknown in the remaining 5 patients. After excluding patients with incomplete data, a history of bariatric surgery, and those taking GLP-1 RA for <3 months, a total of 70 patients were included in the study (Figure 1).

### Baseline characteristics and GLP-1 RA prescription

The baseline characteristics of the study patients are included in Table 1. The mean age was 51.6 ± 12.8 years with 45.7% being female. The mean BMI was 37.1 ± 8.5 kg/m^2^ and 80.1% of the study cohort had obesity; 61.4% had type 2 diabetes mellitus with a mean HbA1c of 8.3% ± 2.2%. Most patients (85.7%) had CHD of moderate to severe complexity and 13 patients (18.6%) had a diagnosis of congestive heart failure, out of which 7 (10% total, 54% of those with heart failure) had heart failure with preserved ejection fraction.

**Table 1:** Baseline Characteristics

Characteristics	Patients on GLP-1 RA (N = 70)
Mean age (y)	51.6 ± 12.8
Sex	
Male	38 (54.3%)
Female	32 (45.7%)
Mean weight (kg)	109.5 ± 25.7
Mean BMI (kg/m^2^)	37.1 ± 8.5
Complexity of congenital heart disease (CHD)^a^	
Simple	10 (14.3%)
Moderate	49 (70%)
Severe	11 (15.7%)
WHO BMI category	
Normal (18.5-24.9 kg/m^2^)	2 (2.9%)
Preobesity (25-29.9 kg/m^2^)	12 (17.1%)
Obesity class I (30-34.9 kg/m^2^)	20 (28.6%)
Obesity class II (35-39.9 kg/m^2^)	13 (18.6%)
Obesity class III (≥40 kg/m^2^)	23 (32.9%)
Diabetes	
Type 2 diabetes mellitus	43 (61.4%)
Type 1 diabetes mellitus	3 (4.3%)
Prediabetes/impaired glucose tolerance	10 (14.3%)
Mean hemoglobin A1c (HbA1c) (%)	8.3 ± 2.2
Congestive heart failure	13 (18.6%)
NYHA functional class	
I	43 (61.4%)
II	18 (25.7%)
III	9 (12.9%)
Presence of prosthetic valve	39 (55.7%)
Systemic ventricle	
Left	67 (95.7%)
Right	3 (4.3%)
Mean systemic ventricular ejection fraction (%)	58.4 ± 8.9
Mean estimated glomerular filtration rate (eGFR) (ml/min/1.73 m^2^)	74.6 ± 16.9
Mean creatinine (mg/dL)	0.93 ± 0.3

Values are mean ± SD or n (%).

BMI = body mass index.

a Based on Warnes CA, Liberthson R, Danielson GK, et al. Task force 1: the changing profile of congenital heart disease in adult life. *J Am Coll Cardio*l. 2001;37(5):1170-5.

Table 2 displays the types of GLP-1 RA prescribed and their dosing. The mean duration of GLP-1 RA use was 21 ± 20 months and 32 patients (45.7%) achieved maximum doses of the respective GLP-1 RA prescribed. Most patients were prescribed semaglutide (65.7%). Six patients (8.6%) were prescribed both semaglutide and liraglutide — all of them were switched from liraglutide to semaglutide due to reasons including cost, availability, insurance approval, or provider preference. Injectable semaglutide was the most frequently prescribed GLP-1 RA, followed by injectable liraglutide, while oral semaglutide was prescribed in approximately 5% of patients.

**Table 2:** GLP-1 RA Prescription Information

Variable	Value
Type of GLP-1 RA prescribed	
Semaglutide	46 (65.7%)
Liraglutide	18 (25.7%)
Both	6 (8.6%)
Subtype of GLP-1 RA used	
Oral semaglutide	4 (5.3%)
Injectable semaglutide (OZEMPIC®)	40 (52.6%)
Injectable semaglutide (WEGOVY®)	8 (10.5%)
Injectable liraglutide (VICTOZA®)	17 (22.4%)
Injectable liraglutide (SAXENDA®)	7 (9.2%)
Initial mean dose	
Injectable semaglutide, mg	0.37 ± 0.24
Oral semaglutide, mg	3 ± 0
Liraglutide, mg	0.7 ± 0.37
End mean dose	
Injectable semaglutide, mg	1.3 ± 0.74
Oral semaglutide, mg	7.7 ± 4.6
Liraglutide, mg	1.8 ± 0.64
Prescriber	
Primary care provider	26 (37.1%)
Endocrinology	23 (32.9%)
Cardiology	13 (18.6%)
Others	4 (5.7%)
Unknown	4 (5.7%)
Reason for prescription	
Weight loss	28 (40%)
Diabetes control	25 (35.7%)
Both	14 (20%)
Unknown	3 (4.3%)
Mean duration of treatment (months)	21 ± 20
Number of patients on maximum doses of GLP-1 RA	32 (45.7%)
Number of patients who discontinued GLP-1 RA	26 (37.1%)
Reason for discontinuation	
High cost	2 (2.9%)
Side effects	8 (11.4%)
Provider changed/discontinued	4 (5.7%)
Unknown/others	12 (17.1%)

Values are n (%) or mean ± SD.

GLP-1 RA = glucagon-like peptide-1 receptor agonist.

Primary care providers were the most frequent prescribers of GLP-1 RA (37.1%) with weight loss being the most common reason for prescription (40%), followed by diabetes control (35.7%). In 20% of the patients, GLP-1 RA was prescribed for both weight loss and diabetes control. The indication for prescription was not available in 3 patients (4.3%). Twenty-six patients (37.1%) discontinued the medication for various reasons (Table 2); 11.4% discontinued due to drug side effects.

### Primary and secondary outcomes

The effects of GLP-1 RA are summarized in Table 3. The mean weight loss of 3.7 kg (95% CI: −5.4 to −1.9 kg; *P* < 0.001) occurred over a mean duration of 21 ± 20 months of GLP-1 RA treatment. Thirty patients (42.9%) achieved ≥5% weight loss compared to their initial weight. The weight loss in these patients ranged between 5% and 18% of their original body weight. BMI improved by 1.2 kg/m^2^ (95% CI: −1.9 to −0.6 kg/m^2^; *P* < 0.001) and HbA1c improved by 0.6% (95% CI [−1.2 to 0]; *P* = 0.054). The mean percentage change in weight was −3.3% (95% CI: −5% to −1.6%; *P* < 0.001), and the mean percentage change in BMI was −3.1% (95% CI: −4.9 to −1.3; *P* < 0.001). There was no statistically significant change in NYHA functional class, eGFR, creatinine, or systemic ventricular ejection fraction.

**Table 3:** Effects of GLP-1 RA Use in Patients With ACHD

	Before GLP-1 RA Use (Mean)	After GLP-1 RA Use (Mean)	Δ Mean	95% CI	*P* Value (Paired *t*-Test)
Body weight (kg)	109.5 ± 25.7	105.8 ± 26.2	−3.7	−5.4 to −1.9	<0.001
BMI (kg/m^2^)	37.1 ± 8.5	35.9 ± 8.5	−1.2	−1.9 to −0.6	<0.001
HbA1c (%)	8.3 ± 2.2	7.7 ± 1.9	−0.6	−1.2 to 0	0.054
eGFR (ml/min/1.73 m^2^)	74.6 ± 16.9	76.8 ± 18.4	2.2	−1.7 to 6.1	0.261
Creatinine (mg/dL)	0.93 ± 0.3	0.98 ± 0.3	0.05	0-0.1	0.016
Systemic ventricular ejection fraction (%)	58.4 ± 8.9	58.8 ± 7.5	0.4	−1.6 to 2.5	0.687

Values are mean ± SD.

ACHD = adult congenital heart disease; BMI = body mass index; eGFR = estimated glomerular filtration rate; GLP-1 RA = glucagon-like peptide-1 receptor agonist; HbA1c = hemoglobin A1c.

The characteristics of the patients who achieved ≥5% weight loss vs those who did not are listed in Table 4. The prevalences of class II and III obesity were the only variables that significantly differed between the 2 groups (*P* = 0.035). For those who were on maximum doses of GLP-1 RA (32 patients, 45.7%), the mean weight loss was 3.1 kg (95% CI: −2.8 to 4.5 kg; *P* = 0.627), and the mean percentage weight loss was 2.78% (95% CI: −2.7% to 4.2%; *P* = 0.652). Among those who stopped taking GLP-1 RA, the mean weight gain was 2.4 kg (95% CI: −2.6 to 7.4 kg; *P* = 0.330) over a mean duration of 36.6 ± 35 months post GLP-1 RA discontinuation.

**Table 4:** Comparative Characteristics of Patients Achieving ≥5% Weight Loss vs Not

	Patients with ≥5% Weight Loss (n = 30)	Patients with <5% Weight Loss (n = 40)	*P* Value
Female	17	15	0.111
Male	13	25	
Initial weight (kg)	108.5 ± 20.8	110.2 ± 28.9	0.788
Initial BMI (kg/m^2^)	37.3 ± 6.5	36.8 ± 9.8	0.810
Initial HbA1c (%)	7.7 ± 1.9	8.2 ± 2.5	0.413
Initial creatinine (mg/dL)	0.96 ± 0.3	0.92 ± 0.2	0.611
Initial eGFR (ml/min/1.73 m^2^)	74.4 ± 19	75.8 ± 14.3	0.744
Type of GLP-1 RA used			0.375
Semaglutide	17	29	
Liraglutide	10	8	
Both	3	3	
Duration of GLP-1 RA use (mo)	21.9 ± 23	20.9 ± 18.4	0.846
Final semaglutide dose (mg)	1.44 ± 0.7	1.16 ± 0.7	0.188
Final liraglutide dose (mg)	1.8 ± 0.7	1.85 ± 0.6	0.843
No. of patients achieving maximum dose of GLP-1 RA	15/30 (50%)	17/40 (42.5%)	0.382
No. of patients with BMI ≥35 kg/m^2^	20/30 (66.7%)	16/40 (40%)	0.035
Type 2 diabetes Mellitus	17/30 (56.67%)	26/40 (65%)	0.306

Values are n, mean ± SD, n/N (%).

BMI = body mass index; eGFR = estimated glomerular filtration rate; GLP-1 RA = glucagon-like peptide-1 receptor agonist; HbA1c = hemoglobin A1c.

By linear regression, there was a statistically significant effect of age on the change in weight (*P* = 0.014), with a 1-year difference in age associated with a 0.17 kg smaller weight change, such that the estimated weight loss for a 30-year-old is 1.7 kg greater than that of a 40-year-old. There was no significant difference in the change in weight by sex.

### Safety endpoints

No patient had renal adverse event defined as >50% reduction in eGFR from baseline. Six patients (8.6%) had fasting hypoglycemia (blood sugar <70 mg/dL), 3 of these 6 patients were insulin-dependent diabetics. There were no major hypoglycemic events requiring corrective action. One patient taking liraglutide required hospitalization due to pancreatitis. Eight patients (11.4%) discontinued the medication due to side effects, the majority of which included gastrointestinal (GI) intolerance. One patient discontinued due to recurrent urinary tract infection and 1 patient developed pancreatitis as mentioned above.

A summary of safety data is mentioned in Table 5. Twenty-three patients (32.9%) developed side effects on GLP-1 RA, most commonly due to GI intolerance (N = 14, 20%), with symptoms including nausea, vomiting, diarrhea, and abdominal fullness.

**Table 5:** Summary of Safety Data

Variable	Number of Patients (%)
Safety endpoints	
Discontinuation due to side effects	8 (11.4%)
Fasting hypoglycemia (fasting blood sugar <70 mg/dL)	6 (8.6%)
Hospitalization due to side effects	1 (1.4%)
Renal adverse event (>50% reduction in eGFR from baseline)	0
Major hypoglycemic event (hypoglycemia requiring corrective action)	0
Number of patients who developed side effects	23 (32.9%)
Side effects	
Gastrointestinal intolerance	14 (20%)
Hypoglycemia (not requiring corrective action)	6 (8.6%)
Recurrent urinary tract infections	1 (1.4%)
Pancreatitis	1 (1.4%)
Headache	1 (1.4%)
Dizziness	1 (1.4%)
Side effects that caused medication discontinuation	
Gastrointestinal intolerance	6 (8.6%)
Pancreatitis	1 (1.4%)
Recurrent urinary tract infections	1 (1.4%)

Values are n (%).

eGFR = estimated glomerular filtration rate.

## Discussion

This is the first study evaluating the effectiveness of GLP-1 RA for the treatment of obesity and diabetes in patients with ACHD. We demonstrate that: 1) GLP-1 RA are safe and well tolerated by most patients with ACHD; 2) GLP-1 RA are effective for weight loss among patients with ACHD, especially younger patients and those with BMI ≥35 kg/m^2^; and 3) GLP-1 RA have beneficial effects on HbA1c in patients with ACHD (Central Illustration).

**Central illustration:**
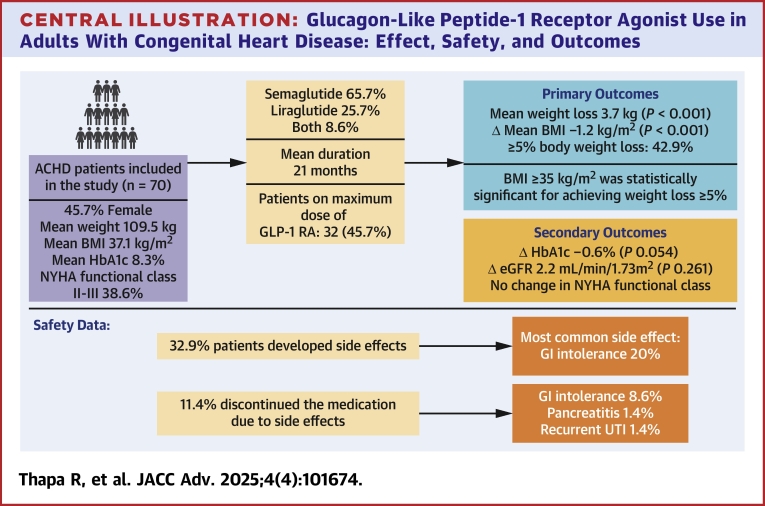
**Glucagon-Like Peptide-1 Receptor Agonist Use in Adults With Congenital Heart Disease: Effect, Safety, and Outcomes** ACHD = adult congenital heart disease; BMI = body mass index; eGFR = estimated glomerular filtration rate; GI = gastrointestinal; GLP-1 RA = glucagon-like peptide-1 receptor agonist; HbA1c = hemoglobin A1c; UTI = urinary tract infection.

The prevalence of obesity in patients with ACHD has been estimated to be as high as 15% to 30%.^3^^,^^16^, ^17^, ^18^, ^19^, ^20^ An adverse cardiovascular risk profile is observed in patients with ACHD with obesity as denoted by worsening hypertension, hyperlipidemia, and type 2 diabetes mellitus.^3^ Obesity also predisposes to obstructive sleep apnea in patients with ACHD^21^ which, in turn, increases arrhythmia and heart failure-associated risks. While an “obesity paradox” has been observed with lower mortality in patients with ACHD with higher BMI,^19^ there is also evidence of increased cardiovascular events with increasing BMI in this population.^3^^,^^22^

In the current study, mean weight loss was 3.7 kg, which is comparable to the weight loss achieved with a lower dose of semaglutide (0.5 mg) in the SUSTAIN (Trial to Evaluate Cardiovascular and Other Long-term Outcomes with Semaglutide in Subjects with Type 2 Diabetes) trials.^23^, ^24^, ^25^, ^26^, ^27^, ^28^ In the STEP (Semaglutide Treatment Effect in People with Obesity)^29^, ^30^, ^31^, ^32^, ^33^, ^34^, ^35^, ^36^ and SELECT (Semaglutide Effects on Cardiovascular Outcomes in People with Overweight or Obesity) trials,^13^ a higher dose of semaglutide of 2.4 mg contributed to a more dramatic change (9%-17%) in total body weight compared to placebo. In randomized clinical trials of liraglutide, mean weight loss ranged between 2 to 8.4 kg.^37^, ^38^, ^39^, ^40^, ^41^, ^42^ While the STEP 8 trial^36^ showed higher average body weight loss with a maximal dose of semaglutide compared to liraglutide, in our study, the type of GLP-1 RA used had no impact on achieving ≥5% weight loss.

Compared to patients with normal BMI, preobesity or class I obesity, patients with ≥class II obesity (BMI of ≥35 kg/m^2^) were more likely to achieve ≥5% weight loss. This is in contrast to previous studies showing weight loss across all groups of BMI.^43^^,^^44^ The weight gain observed following drug discontinuation did not achieve significance in the current study but is recognized to occur in up to two-thirds of patients following discontinuation of GLP-1 RA.^45^

In this study, 61% of patients had type 2 diabetes mellitus with a mean drop in HbA1C of 0.6% treated with a mean maximal semaglutide dose of 1.3 mg and liraglutide dose of 1.8 mg, respectively. This is in contrast to SUSTAIN in which patients with type 2 diabetes mellitus achieved a mean improvement in HbA1C of 1.2% to 1.5% and 1.5% to 1.8% following treatment with 0.5 mg and 1 mg of semaglutide, respectively.^23^, ^24^, ^25^, ^26^, ^27^, ^28^^,^^46^ In LEADER (Liraglutide Effect and Action in Diabetes: Evaluation of Cardiovascular Outcome Results), which examined liraglutide treatment in patients with type 2 diabetes mellitus, HbA1C was lowered by 0.4% to 1.3%.^38^^,^^47^ Among nondiabetics in STEP treated with a higher dose of semaglutide (2.4 mg), the mean HbA1C drop was 0.1% to 0.6%,^29^^,^^31^, ^32^, ^33^^,^^36^ whereas in SCALE (Satiety and Clinical Adiposity - Liraglutide Evidence in Nondiabetic and Diabetic Individuals), a clinical trial of liraglutide treatment of obesity and prediabetes, the mean HbA1C drop was 0.3%.^42^ Taken together, these findings indicate GLP-1 RAs are just as effective for improving glycemic control in patients with ACHD as they are for other at-risk populations.

Side effects are an important limitation of GLP-1 RA treatment. In the current study, just over one-third of patients developed side effects. This is significantly less than the 56% to 96% of patients who developed side effects on various doses of semaglutide/liraglutide in larger studies.^29^^,^^31^, ^32^, ^33^^,^^37^^,^^42^^,^^46^ GI intolerance occurred in 20% of patients, which is again significantly less than the 60% to 80% of patients with GI intolerance in larger GLP-1 RA studies to date.^24^^,^^25^^,^^28^, ^29^, ^30^, ^31^, ^32^, ^33^^,^^36^^,^^39^^,^^40^^,^^48^ A total of 11% of patients stopped the GLP-1 RA due to side effects, which is comparable to prior studies.^10^^,^^29^, ^30^, ^31^, ^32^, ^33^^,^^46^ One patient in the current study developed pancreatitis on liraglutide. In the LEADER and SCALE trials, the incidence of pancreatitis with liraglutide has been reported to be 0.4%.^10^^,^^42^ In the STEP trials, 1 to 3 patients developed pancreatitis on semaglutide.^29^^,^^30^ In the SUSTAIN 6 trial, 3.8% of patients developed nephropathy on semaglutide.^6^ No significant nephropathy was noted in the current study. Similarly, there were no symptomatic or major hypoglycemia events in the current study, although fasting hypoglycemia occurred in 8.6% of patients.

Clinical trials of semaglutide have demonstrated improvement in heart failure symptoms, N-terminal pro-B-type natriuretic peptide, and 6-minute walk distance.^14^^,^^49^, ^50^, ^51^, ^52^, ^53^ In patients with type 2 diabetes mellitus and chronic renal disease, it has been shown to reduce heart failure events and cardiovascular mortality.^54^ It has also been shown to improve left atrial volume and right ventricular dilatation in patients with obesity-related heart failure with preserved ejection fraction.^55^ In our study, we did not observe a significant improvement in group NYHA functional class following GLP-1 RA use. Despite this, we believe that due to the cardiovascular benefits of GLP-1 RA demonstrated in larger clinical trials, it is important to continue these drugs in patients with minimal side effects. In patients unable to tolerate maximal doses, continuing GLP-1 RA at the highest tolerated dose should be considered based on beneficial weight loss still being achieved at submaximal doses.

The findings highlight the therapeutic potential of GLP-1 RA to address a major unmet need for evidence-based treatments for reducing obesity among patients with ACHD, with and without heart failure. More than ever ACHD clinicians need effective strategies for improving functional class, with obesity being a key target given its association with reduced exercise capacity in patients with ACHD.^56^

### Study Limitations

This was a retrospective study, and the number of patients was limited. One of the main limitations is the heterogeneity of the indications, which makes it difficult to compare these data with other studies focusing on obesity or diabetes as the primary indication to start the drug. At the same time, this perhaps depicts the real-world scenario of the use of GLP-1 RA for heterogeneous indications in patients with ACHD.

Tirzepatide, which is currently Food and Drug Administration approved for weight loss, was not included in the current study due to its dual mechanism of action of being both glucagon-like peptide-1 and glucose-dependent insulinotropic polypeptide receptor agonist.

Measured side effects were dependent on documentation by the provider and available laboratory testing, which may not be present in all patients in a “timely” fashion. Since the study was based on data retrieved by retrospective chart review, some variables of interest were missing. Unlike most studies done for GLP-1 RA, the current study does not necessarily include diet/exercise prescriptions. The study limitation also includes the fact that there are no controls. This is a single-center cohort study in a limited number of ACHD patients and one needs to be cautious about applying this to the general ACHD population.

## Conclusions

GLP-1 RAs are safe and effective for weight loss in patients with ACHD with the added benefit of improved glycemic control. Prescription of these agents requires close monitoring for side effects. Clinical trials are needed to demonstrate the clinical potential of GLP-1 RA for improving cardiovascular outcomes in patients with ACHD.

Perspectives **COMPETENCY IN PRACTICE-BASED LEARNING:** GLP-1 RAs are effective for weight loss in patients with adult congenital heart disease, especially those with class II and III obesity. The safety and tolerance of these drugs are acceptable in this population, although some patients may develop side effects leading to drug discontinuation.

**TRANSLATIONAL OUTLOOK:** Obesity has adverse cardiovascular outcomes in adults with congenital heart disease. The findings of this study support the role for GLP-1 RAs in patients with adult congenital heart disease for improved weight loss and glycemic control. However, due to the retrospective nature of the study and small sample size, these results should be verified with future prospective studies with larger number of patients with ACHD.

## Funding support and author disclosures

The authors have reported that they have no relationships relevant to the contents of this paper to disclose>.
